# Detection of Residual Antibiotics and Their Differential Distribution in Broiler Chicken Tissues Using Enzyme-Linked Immunosorbent Assay

**DOI:** 10.3390/antibiotics10111305

**Published:** 2021-10-26

**Authors:** Yasmin El Tahir, Elshafie I. Elshafie, Muhammad Nadeem Asi, Kaadhia Al-Kharousi, Al Ghalya Al Toobi, Yahya Al-Wahaibi, Waleed Al-Marzooqi

**Affiliations:** 1 Department of Animal and Veterinary Sciences, College of Agricultural and Marine Sciences, Sultan Qaboos University, Muscat 123, Oman; yasmin@squ.edu.om (Y.E.T.); eielshafie@squ.edu.om (E.I.E.); asi@squ.edu.om (M.N.A.); kaadhia@squ.edu.om (K.A.-K.); alghalya@squ.edu.om (A.G.A.T.); 2 Ministry of Agriculture & Fisheries, Directorate of Agriculture and Animal Wealth, Muscat 123, Oman; surgeon_xr@hotmail.com

**Keywords:** antibiotic, ELISA, chicken, enrofloxacin, gentamicin, oxytetracycline, sulfamethazine, tylosin

## Abstract

The aim of this study was to estimate the residue levels of five commonly used antibiotics in poultry tissue samples using enzyme linked immunosorbent assay (ELISA). A total of 200 samples that comprised breast and liver (100 each) were collected from five poultry farms randomly selected from Muscat regions. The samples were analyzed for enrofloxacin (ENR), gentamicin (GEN), oxytetracycline (OTC), sulfamethazine (SMZ), and tylosin (TYL) residue concentrations. Comparisons of antibiotic residues between breast and liver of chickens under investigations showed a significant difference of ENR, GEN, OTC, SMZ, and TYL residue concentrations (*p* < 0.05). The highest antibiotic residue concentrations reported in the chicken liver were TYL, GEN, OTC, SMZ, and ENR, respectively. The lowest residual antibiotic concentrations observed in the chicken breast were TYL, GEN, OTC, SMZ, and ENR, respectively. Furthermore, the Kruskal–Wallis statistical test revealed a significant difference between the five antibiotic concentrations in both breast (*H* (4) = 54.69, *p* < 0.05) and liver (*H* (4) = 44.36, *p* < 0.05). A follow up of this finding by Bonferroni correction for both breast and liver samples revealed a significant difference for the breast sample between the concentration of ENR residue, and the concentration of residues for of both OTC and TYL (*p* < 0.05). These data show that not all tissues incorporate antibiotics at the same concentration. The results of this study could support regulatory bodies in adopting, monitoring, and enforcing guidelines pertinent to safety levels of different antibiotic residue concentrations in poultry meat when antibiotics are used for different indications.

## 1. Introduction

There is growing concern over the use of antibiotics in livestock production in the Middle East countries, including Oman. Other countries such as the USA and countries in Europe have prevented such use, except for indicated treatment of diseases, considering that uncurbed use of antibiotics is a major problem in food safety. These tally with the fact that antibiotic residues are key drivers of antibiotic resistance, with consequent risk to human health. In practice, antibiotics can be used for treatment and prevention of several diseases, as well as for improving feed efficiency, promoting growth, and most important, for preventing economic losses to growers [1,2,3], as is the case in the Middle East and some other countries. A standard measure of residual antibiotic concentration is the maximum residual limit (MRL), defined as the maximum concentration of an antibiotic or chemical permitted acceptable in a diet for human or animal consumption [4]. These limits have been defined by competent authorities to limit the dangers of antibiotic use in animal products entering the food chain. Though no longer in use for poultry in many countries, but still used in some countries, fluoroquinolones (ciprofloxacin, enrofloxacin, danofloxacin, sarafloxacin) are used as broad spectrum antimicrobial agents against Pseudomonas spp. The issue with fluoroquinolones is their longer elimination half-life in poultry than in mammals [5] and the subsequent development of bacterial resistance for such critical antibiotics [6]. Other researchers [7] reported that when using both the lowest and highest food and drug administration approved doses of enrofloxacin, breast tissue had consistently higher drug concentrations than thigh tissues during the dosing period.

Antibiotics pose a threat to consumer health, such as allergy, gastrointestinal disorders, and development of bacterial resistance. Antibiotics can penetrate and remain in an animal’s tissues, especially if the initial antibiotic dosage is high or withdrawal time is not sufficient before slaughtering [8,9]. There are several antibacterial agents that are no longer or are very rarely used in poultry production, including the aminocyclitols (e.g., apramycin, spectinomycin) and amphenicols (e.g., florfenicol). Similarly, chloramphenicol is prohibited from use in any food producing species, including poultry [10]. Aminoglycosides including gentamicin, are typically only used in the hatchery by oral or subcutaneous injection at a one day of age and therefore do not pose a risk with respect to residues in meat at processing.

Macrolides (erythromycin, roxithromycin, spiramycin, tilmicosin, tylosin) are bacteriostatic antimicrobial agents. Macrolides are metabolized in the liver, and the highest tissue concentrations for chickens and turkeys are also found in the liver [11]. Tylosin and spiramycin have been studied in growing chicks and results show that tylosin residues are not detected more rapidly in the liver than spiramycin residue. Sulfonamides are bacteriostatic antibacterial agents approved for use in food-producing animals, but human consumption of sulfonamide-contaminated products can cause central nervous system effects, gastrointestinal disturbances, and hypersensitivity reactions [12]. Tetracyclines (oxytetracycline, chlortetracycline, doxycycline, and tetracycline) are broad spectrum antibacterial agents used in the treatment of avian infectious diseases, especially in bacterial respiratory tract diseases [13].

Several screening and confirmatory analytical methods for the detection of antibiotic residues have been developed [14]. The immunoassay assay method has been widely used in the evaluation of antimicrobial residues in the food chain [15]. For example, Ramatla et al. [15] evaluated different methods for monitoring antimicrobial residues in meat samples. They concluded that the enzyme-linked immunosorbent assay (ELISA) method provided excellent sensitivity and selectivity for the determination of antibiotic residues in meat samples, and therefore, in this study, we opted for the ELISA method. This study was designed to determine the residual amounts of antibiotics commonly used in poultry farms in Muscat region in the Sultanate of Oman.

## 2. Methods

### 2.1. Ethical Consideration

The study was approved by the Animal Research Ethics Board at Sultan Qaboos University (Ethical Code: RF/AGR/ANVS/18/04). All experimental work was conducted at the Poultry Research Unit at the Agricultural Experiment Station in accordance with the experimental unit policy on animal welfare, and the requirements of the procedures involving animals/birds and their care were conducted in conformity with international laws and policies (EEC Council directives 86/609, OJL 358, 1 December, 12, 1987; NIH Guide for the Care and Use of Laboratory Animals, NIH Publications No. 85-23, 1985) at Sultan Qaboos University

### 2.2. Antibiotics Competitive Enzyme Immunosorbent Assay Kits

The antibiotic competitive enzyme immunosorbent assay kits were purchased from Shenzhen Lvshiyuan Biotechnology Company, China. SMZ (catalog no. LSY-100-16), ENR (catalog no. LSY-10017), TYL (catalog no. LSY-10020), GEN (catalog no. LSY-10023), and OTC (catalog no. LSY 10014). Each kit was specified for a different antibiotic. Two extractions were performed for each sample. Sensitivity, detection limit, and recovery rate for each kit were as follows: SMZ (sensitivity 1 ppb, detection limit 5 ppb, and recovery rate 85 ± 25%); ENR (sensitivity 1 ppb, detection limit 1 ppb, and recovery rate 80 ± 15%); TYL (sensitivity 1 ppb, detection limit about 1 ppb, and recovery rate 90 ± 30%); GEN (sensitivity 0.05 ppb, detection limit 2.5 ppb for tissue and 3 ppb for liver, recovery rate 90 ± 20% for tissue and 80 ± 20% for liver); OTC (sensitivity 0.4 ppb, detection limit about 20 ppb, and recovery rate 90 ± 15%). Each immunoassay kit contained sufficient material for 96 measurements. Each microtiter consisted of 96 wells, coated with capture antibodies against each antibiotic.

### 2.3. Sample Collection

Given the frame and plan of the study and facilities available, it was not feasible at the time of collection to gather detailed information relevant to management in each farm, noting that all just aimed at marketing chicken at a certain age.

Five privately owned poultry farms from the Muscat region were randomly selected for the study. A total of 100 live birds (20 birds from each farm) were collected in the study area and sacrificed by standard slaughtering process to collect a total of 200 samples, which comprised breast and liver, 100 each. Each sample was placed into a separate plastic zipper bag, numbered, and then transferred to the laboratory in an iced plastic container and stored at −20 °C until extraction. The samples were subjected later to grinding before the extraction procedure.

### 2.4. Extraction Procedure

For extraction, samples were homogenized according to the manufacture instruction, as we followed the procedure specified for each antibiotic kit (Protocol for detection of antibiotic residues in Supplementary Materials). Briefly, the tissue samples (breast and liver) were homogenized by using an ultra-meat homogenizer, i.e., without any buffer. This was subsequently followed by a sample of one to two grams of tissue homogenate (as specified in each kit) being weighed in centrifuge tube and adding 1 mL of N, N Dimethyl formamide (DMF) to that. After shaking, the resultant homogenate was centrifuged at 4000 rpm at room temperature for 10 min. An amount of 100 µL of the supernatant was taken and 900 µL of the diluted dissolving solution was added to it. An amount of 50 µL of the dissolved supernatant was taken for analysis. Two extractions were carried out for each sample.

### 2.5. Competitive Enzyme Linked Immunosorbent Assay

For this test, we followed the procedure of the manufacturer for each antibiotic kit. Briefly, a volume of 50 µL of each standard solution or prepared sample was added to each well, followed by 50 µL of anti-antibiotic solution. The plates were incubated at room temperature for 1 h, and then washed three times with 250 µL of washing buffer, followed by the addition of 100 µL of enzyme conjugate to each well. After incubation for 15 min, the plates were washed three times with 250 µL of washing buffer. Then, 50 µL of the substrate and 50 µL of chromogen were added to each well and incubated in the dark for 15 min at room temperature. Finally, 100 µL of stop solution was added to stop the reaction, and absorbance was recorded at 450 nm within 30 min. A calibration curve was plotted for standard concentration and OD. The mean values of the absorbance values were calculated as follows: percentage of absorbance value = B/B_0_ × 100 %, where B is the average OD value of the sample or the standard solution, B_0_ is the average OD value of the 0 ng/mL standard solution.

### 2.6. Statistical Analysis

The data were analyzed statistically using IBM SPSS^®^ Statistics 20. Since the data were not normally distributed, the Mann–Whitney U test with a level of significance set up at *p* < 0.05 was performed to compare the antibiotic residue concentrations in breast muscle and liver tissues. The Kruskal–Wallis test with the same level of significance was used to test statistical significance among the five detected antibiotic residue concentrations. Further analysis using the post hoc Mann–Whitney test was applied to follow up the findings of the Kruskal–Wallis test. The Bonferroni correction was performed to control type one error, and so all effects of dummy variables were reported at a 0.005 (0.05/number of comparisons) level of significance.

## 3. Results

The ELISA technique was used to detect different antibiotic residue concentrations in breast and liver of broiler chickens. The quantity of antibiotic residue concentrations in broiler meat and liver samples are presented in Table 1. In general, findings of the current study indicate that samples contained various levels of residues of different antibiotics (Table 1). Gentamicin residue concentrations were detected at a higher level in breast in comparison with other antibiotics, whereas in liver, both oxytetracycline and sulfamethazine residue concentrations were the highest detected antibiotics.

### 3.1. Comparison of Antibiotic Residues Detected in Broiler Chicken Tissues

The Mann–Whitney test (based on mean rank and alpha of 0.05) was used to assess the significance of differences among broiler chicken tissues; i.e., breast and liver. A significant difference between enrofloxacin, gentamicin, oxytetracycline, sulfamethazine, and tylosin residue concentrations were observed between the breast and liver samples (*p* < 0.05; Table 2). The lowest residual antibiotic concentrations observed in the chicken breast were tylosin, gentamicin, oxytetracycline, sulfamethazine, and enrofloxacin, respectively. However, the highest antibiotic residue concentrations reported in the chicken liver were tylosin, gentamicin, oxytetracycline, sulfamethazine, and enrofloxacin, respectively. (Table 2).

### 3.2. Differences in Concentration of Antibiotic Residues Recovered from Broiler Chickens Breast and Liver Tissues

The Kruskal–Wallis test at alpha 0.05 significance was applied to reveal statistically significant differences among the five detected antibiotic residues in broiler chicken tissues (Table 3). Overall, five of the antibiotic residues detected from the chicken breast and liver were statistically significant, as indicated by the Kruskal–Wallis test (*H* (4) = 54.69, *p* < 0.05) and (*H* (4) = 44.36, *p* < 0.05), respectively (Table 3).

Mann–Whitney tests were used to follow up the significance among the five detected antibiotic residues in broiler chicken tissues, and a Bonferroni correction was applied as well, so all effects are reported at a 0.005 level of significance (Table 4). In breast muscle, there was a significant difference between enrofloxacin residue concentration and the residues of both oxytetracycline and tylosin (*p* < 0.05). The difference between tylosin residue concentration and concentrations of both gentamicin and sulfamethazine residues was significant (*p* < 0.05), with tylosin being lower. However, there was no significant difference between other residue antibiotic concentrations (*p* > 0.05) (Table 4).

In liver tissue, there was a significant difference between the tylosin residue concentration and the residue concentrations of enrofloxacin, gentamicin, and sulfamethazine (*p* < 0.05). The sulfamethazine residue concentration was also significant compared with that of gentamicin (*p* < 0.05). However, there was no significant difference between the other residue antibiotic concentrations (*p* > 0.05) (Table 4).

## 4. Discussion

The poultry industry is a growing sector in Oman, with several brands and companies having been launched in recent years. There are large corporate producers, but as well there are small- and medium-size farms scattered around the country, readily supplying consumers. Poultry feed constitutes a major cost component in production, and feed suppliers in the Middle East incorporate varying levels of antibiotics as supplements to enhance growth and for prophylaxis. Therefore, this study represents an overarching stage to counteract the bacterial resistance resulting from some antibiotic residues in poultry meat, and also an attempt to better align with standards pertaining to human health. It should also be noted that our study reflects the prevailing characteristics of chicken meat in the local market.

To probe this, we sampled chicken for this study based on a diligent search for information on use of antibiotics by farmers in their privately owned farms. Nonetheless, the presence of antibiotics, whether in water or feed, could hardly be ascertained since farmers here just collectively acknowledged obtaining feed that was prevailing in the market, with no additional treatments throughout the growing period. This should be considered since competitiveness dictates taking into account the additional cost of antibiotics incurred by farmers in that region, should they apply antibiotics on their own. Moreover, this study is the first that paves the way into further investigation of food safety and the presence of residual antibiotics in poultry products in Oman, as well as raising awareness of the indiscriminate use of antibiotics in food producing species. Pertinently, the chickens from all farms used in the study were all about the same slaughter age (about 7 weeks of age), which is important for the assessment of the level of antibiotics that would be indicative of a threshold for safe consumption of such products, given the impact of the residual effect of antibiotics on the emergence of antibiotic-resistant bacterial strains. It also sets the stage for regulatory bodies to reconsider and enforce MRL in the country that are in line with procedures that benefit public health.

The present study, using ELISA, showed that samples from the liver and breast of broiler chicken, these being organoleptically favored in Middle East countries, contain various levels of residues of different antibiotics. Antibiotics can penetrate and remain in an animal’s tissues, especially if the initial dosage of the antibiotic is high or if the withdrawal time is not sufficient before slaughtering [8].

In this study, ENR residues in both breast and liver samples from farm five were above the MRL. ENR, a kind of amphoteric of the quinolone (fluoroquinolone) antimicrobial family, is a potent antibiotic with a broad spectrum of activity against Gram-positive and Gram-negative micro-organisms [1]. Quinolones may have a direct effect through inhibiting bacterial DNA-gyrase and topoisomerase IV enzyme activities or lead to the emergence of drug-resistant bacteria, predicating a potential risk to human health [16]. The use of quinolone as prophylaxis and treatment of chicken and beef is legal in some countries. It is worth mentioning that all the farms used in this study were privately owned. Therefore, from the results obtained, it is necessary to monitor frequently consumed animal products with high nutritional value for the presence of residues of quinolones in Oman. There is a need for a limit and strict surveillance in the applied dose and observation of pre-slaughter withdrawal times.

Although the residual levels of GEN, OTC, SMZ, and TYL in both breast and liver samples were below the MRL, their hazard for human health still exists. In this study TYL residue concentrations were the highest recovered from liver (mean rank 150.33) compared with breast (50.67). Our results are nearly consistent with Mossad et al. [17], in which they used tilmicosin (a macrolide antibiotic synthesized from TYL). Tilmicosin could not be detected by microbiological assay in all tested tissues except in lung, liver, and kidneys. However, due to financial limitations in this study, we excluded other tissues and only opted for liver and breast tissues. TYL is a macrolide antibiotic that is active against certain Gram-positive and Gram-negative bacteria, especially different members of Mycoplasma spp. Tylosin is registered exclusively for veterinary use in several countries, primarily for use in the chronic respiratory disease complex in chickens and infectious sinusitis in turkeys. In some countries, TYL is registered for use as a growth promoter for poultry, pigs, and cattle [18].

It is well recognized that the steps in food processing should be kept under continuous monitoring for preventing overexpressed levels of drug residues. This work was a prelude to unraveling the extent of residual antibiotics in poultry chicken in Oman. As it revealed residual antibiotic levels of a number of antibiotics in chicken meat samples from randomly selected farms, it must be a reminder to heed the recommended use of antibiotics, lest the risk of development of antibiotic resistance should escalate. This should encourage regulatory bodies to adopt and enforce guidelines for antibiotics use in poultry based on specifically identified indications. This study furnishes the base for further studies on the regulated use of veterinary drugs in Oman.

## 5. Conclusions

The results indicate the presence of antibiotic residues and that ENR residue concentrations were above the MLR. The consumption of this contaminated meat can result in antibiotic resistance in consumers and poses a public health threat. In Oman, most of the privately owned poultry farms and farmers do not engage the services of veterinarians for disease diagnosis and drug prescription. They claim to recognize disease conditions based on their knowledge and experience of clinical signs and postmortem findings. Therefore, there is a need to educate farmers and encourage them to seek laboratory confirmation of disease diagnosis and to conduct antibiotic sensitivity tests for bacterial isolates. National authorities should also adopt more judicious approaches to ensure the prudent use of antibiotics in food animals.

## Figures and Tables

**Table 1:** Minimum and maximum range with median (in µg/kg) of different antibiotic residue concentrations in breast and liver of broiler chickens from five farms.

Antibiotics	Minimum and Maximum Range of Antibiotic Residues (Median)	
	Breast	Liver
Enrofloxacin	0.08–499.22 (0.54)	0.04–477.37 (1.87)
Gentamicin	0.00–161.40 (1.13)	0.20–42.50 (1.78)
Oxytetracycline	0.00–71.18 (0.00)	0.00–147.48 (8.18)
Sulfamethazine	0.07–38.48 (0.79)	0.00–147.48 (8.18)
Tylosin	0.00–2.22 (0.23)	1.26–15.69 (7.53)

Maximum residue limit (MRL) in liver and breast are 200 µg/kg and 100 µg/kg, respectively, for Enrofloxacin; 100 µg/kg and 100 µg/kg, respectively, for gentamicin, sulfamethazine, and tylosin; 600 µg/kg and 200 µg/kg, respectively, for oxytetracycline.

**Table 2:** Mean rank comparison of antibiotic residue concentrations (µg/kg) between breast and liver tissues of broiler chickens using Mann–Whitney U test.

	Antibiotic Residues (Mean Rank)		Mann–Whitney U	*p* value
Antibiotics	Breast	Liver		
Enrofloxacin	91.10	109.90	4060.00	0.020
Gentamicin	77.61	123.40	2710.50	0.001
Oxytetracycline	88.25	112.76	3774.50	0.001
Sulfamethazine	90.14	110.86	3964.00	0.011
Tylosin	50.67	150.33	17.00	0.001

**Table 3:** Comparison of the mean rank between the five antibiotic residue concentrations (µg/kg) detected in each tissue of liver and breast of broiler chicken using Kruskal–Wallis test.

Tissues	Mean Rank	Kruskal–Wallis	Degrees of Freedom	*p* Value
**Breast**				
Enrofloxacin	275.55	54.69	4	0.001
Gentamicin	305.82			
Oxytetracycline	222.67			
Sulfamethazine	276.81			
Tylosin	171.66			
**Liver**				
Enrofloxacin	245.52	44.36	4	0.001
Gentamicin	241.90			
Oxytetracycline	253.22			
Sulfamethazine	188.52			
Tylosin	323.36			

**Table 4:** Comparisons of antibiotic residue concentrations (µg/kg) among different organs of chicken by Mann–Whitney tests using Bonferroni correction at a 0.005 level of significance.

Antibiotic Residues	*p* Value			
	Gentamicin	Oxytetracycline	Sulfamethazine	Tylosin
**Breast**				
Enrofloxacin	0.036	0.001 *	0.401	0.001 *
Gentamicin	-	0.084	0.015	0.001 *
Oxytetracycline	0.084	-	0.048	0.601
Sulfamethazine	0.015	0.048	-	0.001 *
Tylosin	0.001 *	0.601	0.001 *	-
**Liver**				
Enrofloxacin	0.429	0.221	0.225	0.004 *
Gentamicin	-	0.752	0.001 *	0.001 *
Oxytetracycline	0.752	-	0.082	0.832
Sulfamethazine	0.001 *	0.082	-	0.001 *
Tylosin	0.001 *	0.832	0.001 *	-

* Significant difference.

## Data Availability

The data presented in this study are available on request from the corresponding author. The data are not publicly available due to data protection regulations.

## Supplementary Materials

The following are available online at https://www.mdpi.com/article/10.3390/antibiotics10111305/s1, Supplementary materials: This protocol showed good sensitivity and detectability as we used it for detecting residues of different antibiotics. File1: PDF title: Protocol for detection of antibiotic residues.
