# Supplementary material for: Detection of Residual Antibiotics and Their Differential Distribution in Broiler Chicken Tissues Using Enzyme-Linked Immunosorbent Assay
Source: Antibiotics (Basel). 2021 Oct 26;10(11):1305. doi: 10.3390/antibiotics10111305 (PMC8614859; doi:10.3390/antibiotics10111305)
Supplement: Supplementary file 1 [file antibiotics-10-01305-s001.zip › antibiotics-1408190-supplementary.pdf]

Enrofloxacin ELISA Kit

Catalog No. LSY-10017

1. Principle

This test kit is based on the competitive enzyme immunoassay for the detection of Enrofloxacin in samples. The coupling antigens are pre-coated on the micro-well stripes. The Enrofloxacin in the sample and the coupling antigens pre-coated on the micro-well stripes compete for the anti-Enrofloxacin antibodies. After the addition of the enzyme conjugate, the TMB substrate is added for coloration. The optical density (OD) value of the sample has a negative correlation with the Enrofloxacin in the sample. This value is compared to the standard curve and the Enrofloxacin concentration is subsequently obtained.

2. Technical specifications

Sensitivity: 1 ppb

Incubation Temperature: 25℃

Incubation Time: 30min—15min

Detection limit

Tissue, egg..1 ppb

Serum, honey..2 ppb

Cross-reaction rate:

Enrofloxacin..100%

Recovery rate:

Tissue, egg, serum..80±15%

Honey..75±15%

3. Components

1） Micro-well strips: 12 strips with 8 removable wells each
2） 6× standard solution (1 mL each): 0ppb, 1ppb, 3ppb, 9ppb, 27ppb and 81ppb
3） Enzyme conjugate (7ml) ....red cap
4） Antibody working solution (7 mL)..blue cap
5） Substrate A solution (7 mL)..white cap
6） Substrate B solution (7 mL)..black cap
7） Stop solution (7 mL)..yellow cap
8） 20× concentrated washing buffer (40 mL)..white cap
9） 2× concentrated redissolving solution (50 mL)..transparent cap

4. Materials required but not provided

1) **Equipments:** microplate reader, printer, homogenizer, nitrogen-drying device, vortex, centrifuge, measuring pipets, balance (a sensibility reciprocal of 0.01 g)
2) **Micropipettors:** single-channel 20-200 μL, 100-1000 μL, and multi-channel 250 μL;
3) **Reagents:** HCl, Methylene chloride, Acetonitrile (CH_3CN), N-hexane, $Na_2HPO_4 \cdot 12H_2O$, $NaH_2PO_4 \cdot 2H_2O$, Heparin sodium

5. Sample pre-treatment

Instructions (The following points must be dealt with before the pre-treatment)

1) This test kit can detect tissue sample: animal tissue, poultry, aquatic. Eg: Chicken, duck, bovine, rabbit, fish, shrimp etc.
2) Only the disposable tips can be used for the experiments and the tips must be changed when used for absorbing different reagents;
3) Before the experiment, each experimental utensil must be clean and should be re-cleaned if necessary, in order to avoid the contamination that interferes with the experimental results.

Solution preparation before sample pre-treatment:

1) 0.1 M HCl: 860μl HCl (36%) + deionized water 100 mL.
2) Acetonitrile (CH_3CN) -Methylene chloride mixing solution
   $V_{acetonitrile}$-$V_{methylene\ chloride}$ = **1 : 4**
3) pH7.2 0.02M PB buffer: dissolve 5.16 g $Na_2HPO_4 \cdot 12H_2O$ + 0.87 g $NaH_2PO_4 \cdot 2H_2O$ in the deionized water to 1 L.
4) Acetonitrile (CH_3CN)- Methylene chloride- 0.1 M HCl mixing solution
   100ml Acetonitrile (CH_3CN) -Methylene chloride mixing solution (**$V_{acetonitrile}$-$V_{methylene\ chloride}$ = 4 : 1)**, add 5ml 0.1 M HCl solution.
5) The 2× concentrated redissolving solution is diluted with deionized water at 1:1 (1 mL concentrated redissolving solution + 1 mL deionized water), used for the sample redissolving.

5.1 Tissues (chicken/liver, pork/liver, fish, shrimp etc.), egg

1) Weigh 2.0 ± 0.05 g of the homogenized tissue sample or egg sample into 50 ml centrifuge tube
2) Add 8 ml of the Acetonitrile (CH_3CN) -Methylene chloride mixing solution, shake for 5 min, centrifuge at above 4000 r/min at 15 ℃ for 10 min
3) Take 4 ml the clear organic phase into a dry tube, blow to dry with nitrogen or air completely by rotary evaporation at 56 ℃
4) Dissolve the dry residues in 1 mL of the diluted redissolving solution, add 1 mL N-hexane, mix for 30 seconds; centrifuge at above 4000 r/min at 15℃ for 5 min.
5) Remove the upper layer, take 50μl lower layer solution for further analysis.
   Fold of dilution of the sample: 1

5.2 Serum

1) Use centrifuge tube with heparin sodium (20-30 unit/ml blood) to collect chicken blood sample (Suggestion: blood collection syringes are recommended rinsing with heparin).Place the blood sample in the room temperature for 1 hour. After obtain plasma, centrifuge at above 4000r/min at 15 ℃ for 10 min, take out 1 ml plasma.
2) Add CH_3CN (without water) 4 ml , mix up-and-down thoroughly for 5 min, centrifuge at above 4000r/min at 15 ℃ for 10 min.
3) Move the clear supernatant (upper layer) to another centrifuge tube, add 2ml 0.02M PB buffer, mix evenly.
4) Add 5 ml Methylene chloride, mix evenly for 5 min, centrifuge at above 4000r/min at 15 ℃ for 10 min, remove the upper layer, take the lower organic phase to dry bottle(clear without impurities), blow to dry with nitrogen or air completely by rotary evaporation at 50 ℃
5) Dissolve the dry residues in 1 mL of the diluted redissolving solution, add 1 mL N-hexane, mix for 30 seconds; centrifuge at above 4000 r/min at 15℃ for 5 min.

6) Absorb out lightly the upper and middle layer white impurities, take lower phase 100μl, add 100μl diluted redissolving solution, mix for 30s.
7) Take 50μl solution for further analysis.

Fold of dilution of the sample: 2

5.3 Honey

1. Weigh 2.0 ± 0.05g honey sample into 50ml centrifuge tube, add 8 ml Acetonitrile (CH_3CN)-Methylene chloride- 0.1 M HCl mixing solution, shake fully for 3min, centrifuge at above 4000 r/min at 15 ℃ for 10 min.
2. Take 2ml the supernatant (upper layer), blow to dry with nitrogen or air at 56 ℃.
3. Add 1 mL the diluted redissolving solution, shake fully for 1min.
4. Take 50 μL for further analysis

Fold of dilution of the sample: 2

6. ELISA procedures

6.1 Instructions

1. Bring all reagents and micro-well strips to the room temperature (20-25 ℃) before use;
2. Return all reagents to 2-8 ℃ immediately after use;
3. The reproducibility of the ELISA analysis, to a large degree, depends on the consistency of plate washing. The correct operation of plate washing is the key point in the procedures of ELISA;
4. For the incubation at constant temperatures, all the samples and reagents must avoid light exposure, and each microplate should be sealed by the cover membrane.

6.2 Operation procedures

1. Take out all the necessary reagents and place at the room temperature (20-25 ℃) for at least 30min. Note that each reagent must be shaken to mix evenly before use;
2. Take the required micro-well strips and plate frames. Re-sealed the unused microplate, stored at 2- 8 ℃;
3. Solution preparation: dilute 40 mL of the 20× concentrated washing buffer with the deionized water at 1:19 (1 part of 20× concentrated washing buffer + 19 parts of deionized water), or prepare as quantity needed;
4. Numbering: number the micro-wells according to samples and standard solution; each sample and standard solution should be performed in duplicate; record their positions;
5. Add 50 μL of the sample or standard solution to separate duplicate wells, add 50 μL of enzyme conjugate, then add 50 μL of the antibody working solution into each well. Mix by shaking gently, seal the microplate with the cover membrane, and **incubate at 25℃ for 30min;**
6. Wash the microplate with the washing buffer at 250 μL/well for four to five times.;soak the well with the washing buffer for 15-30 sec, flap to dry with absorbent paper (if there are the bubbles after flapping, cut them with the clean tips);
7. Coloration: add 50 μL of the substrate A solution and 50 μL of the B solution into each well. Mix by shaking gently, and **incubate at 25 ℃ for 15 min in the dark for coloration;**
8. Determination: add 50 μL of stop solution into each well. Mix by shaking gently. Set the wavelength of the microplate reader at 450 nm to determine the OD value. (Recommend to

read the OD value at the dual-wavelength 450/630 nm within 5 min) .

7. Result judgment

There are two methods to judge the results; the first one is the rough judgment, while the second is the quantitative determination. Note that the OD value of the sample has a negative correlation with the content of Enrofloxacin in the sample.

7.1 Qualitative determination

The concentration range (ng/mL) obtained from the comparison the average OD value of the sample with that of the standard solution. Assuming that the OD value of the sample I is 0.238, and that of the sample II is 0.946, the OD value of standard solutions is: 1.845 for 0 ppb, 1.542 for 1 ppb, 1.130 for 3 ppb, 0.635 for 9 ppb, 0.326 for 27 ppb ,0.156 for 81 ppb, accordingly the concentration range of the sample I is 27 to 81 ppb, and that of the sample II is 3 to 9 ppb. (multiplied by the corresponding dilution fold)

7.2 Quantitative determination

The mean values of the absorbance values is equivalent to the percentage of the average OD value (B) of the sample and the standard solution divided by the OD value (B_0) of the first standard solution (0 standard) and subsequently multiplied by 100%, that is,

$$\text{Percentage of absorbance value} = \frac{B}{B_0} \times 100\%$$

B—the average (double wells) OD value of the sample or the standard solution

B_0—the average OD value of the 0ng/mL standard solution

Draw the standard curve with the absorption percentages of the standard solutions and the semilogarithm values of the Enrofloxacin standard solutions (ng/mL) as Y- and X-axis, respectively. Read the corresponding concentration of the sample from the standard curve by incorporating its absorption percentage into the standard curve. The resulting value is subsequently multiplied by the corresponding dilution fold, finally obtaining the Enrofloxacin concentration in the sample.

Using the professional analyzing software of this kit will be more convenient for the accurate and rapid analysis of a large amount of samples. (Please contact us for this software)

8. Precautions

1. The room temperature below 25 ℃ or the temperature of the reagents and the samples being not returned to the room temperature (20-25 ℃) will lead to a lower standard OD value.
2. Dryness of the microplate in the washing process will be accompanied by the situations including the non-linear standard curves and the undesirable reproducibility; So continue to next step immediately after washing.
3. Mix evenly, otherwise there will be the undesirable reproducibility.
4. The stop solution is the 2 M sulfuric acid solution, avoid contacting with the skin.
5. Do not use the kit exceeding its expiry date. The use of diluted or adulterated reagents from the kits will lead to the changes in the sensitivity and the detecting OD values. Do not exchange the reagents from the kits of different lot numbers to use.
6. Put the unused microplate into an auto-sealing bag to re-seal it. The standard substance and the colourless color former is light sensitive, and thus they cannot be directly exposed to the

light.

7. Discard the colouration solution with any color that indicates the degeneration of this solution. The detecting value of the 0 standard solution of less than 0.5 (A450 nm< 0.5) indicates its degeneration.
8. The optimum reaction temperature is 25 ℃, and too high or too low temperatures will result in the changes in the detecting sensitivity and OD values.

9. Storage and expiry date

Storage: store at 2-8 ℃, not frozen.

Expiry date: 1 year; date of production is on box.

Note: If the Vacuum package of microplate has leakage, it is still valid to use, do not affect the test result, be relax to use.

Shenzhen Lvshiyuan Biotechnology Co., Ltd
D Building, National Biological Industrial Park of Marinelife, No.2 Binhai Road, Dapeng, Shenzhen, 518120 China
Tel. 86-755-28438788 Fax 86-755-28938800
Email: info@lsybt.com
www.lsybt.com

Oxytetracyline ELISA Test Kit

Catalog No. LSY-10044

1. Principle

This test kit is based on the competitive enzyme immunoassay for the detection of Oxytetracyline in the sample. The coupling antigens are pre-coated on the micro-well stripes. The Oxytetracyline in the sample and the coupling antigens pre-coated on the micro-well stripes compete for the anti-Oxytetracyline antibody. After the addition of the enzyme conjugate, the TMB substrate is added for coloration. The optical density (OD) value of the sample has a negative correlation with the Oxytetracyline in it. This value is compared to the standard curve and the Oxytetracyline concentration is subsequently obtained.

2. Technical specifications

Sensitivity: 0.4 ppb

Incubation Temperature: 25℃

Incubation Time: 30min—15min

Detection limit:

Tissue, Honey..about 20ppb

Milk (method 1)..about 20ppb

Milk (method 1)..about 40ppb

Feed..about 600ppb

Note: ppb=ng/mLor ng/g

Cross-reaction rate:

Oxytetracyline..100%

Tetracycline..200%

Chlortetracycline..240%

Doxycycline..20%

Recovery rate: 90±15%

3. Components

1） Micro-well strips: 12 strips with 8 removable wells each

2） 10× concentrated standard solution: 0 ppb, 4ppb, 12ppb, 36ppb, 108ppb (0.5ml/bottle)

3） Enzyme conjugate (7 mL)

4） Antibody working solution (7 mL)

5） Substrate A (7 mL)

6） Substrate B (7 mL)

7） Stop solution (7 mL)

8） 20× concentrated washing buffer (30 mL)

9） 20× concentrated redissolving solution (10 mL)

4. Materials required but not provided

1) **Equipments**: microplate reader (450nm, 630nm), rotary evaporator/nitrogen-drying device, homogenizer, oscillator, centrifuge (4000g and above), balance (a sensibility reciprocal of 0.01

g), measuring pipets, incubator (adjustable 25℃、37℃、60℃),timer

2) **Micropipettors**: single-channel 20~200 μL and 100~1000 μL, and multi-channel 30~300 μL;
3) **Reagents**: deionized water, HCl, N,N-Dimethylformamide(DMF)

5. Sample pre-treatment

Instructions

The following points must be dealt with before the pre-treatment of any kind of sample:

1) Only the disposable tips can be used for the experiments and the tips must be changed when used for absorbing different reagents;

2) Before the experiment, each experimental equipment must be clean and should be re-cleaned if necessary, in order to avoid the contamination that interferes with the experimental results.

Solution preparation before sample pre-treatment:

1) Dilute 20× concentrated redissolving solution with deionized water at 1:19(1 part concentrated redissolving solution + 19 parts deionized water).
2) Washing buffer: 1 part 20× concentrated washing buffer + 19 parts deionized water
3) 1M HCl: take 1ml concentrated HCl, add 11ml deionized water to dissolve and mix it evenly.

5.1 Tissue

1. Take 1± 0.01 g of the homogenized sample into 10 mL centrifuge tube, add 1 mL N,N-Dimethylformamide(DMF), shake with oscillator for 5min, to make sample completely dispersed, fully contact with the organic phase.
2. Centrifuge at above 4000g for 10 minutes.
3. Take 100ul up-layer clear liquid, add 900ul diluted redissolving solution, shake with oscillator for 10min;
4. Take 50 μL for analysis.

Fold of dilution of the sample: 20

5.2 Honey

1) Take 1± 0.01g honey sample into 10 mL centrifuge tube; Add 2ml deionized water, shake with oscillator fully for 1 min to dissolve; take 100ul dissolved solution, add 400ul diluted redissolving solution, oscillator for 10S to mix it evenly;

2) Take 50ul for analysis immediately.

Fold of dilution of the sample: 10

5.3 Milk (method 1)

1) Thaw the collected liquid milk sample, then put at room temperature for 30min;

2) Put tips in the down-layer of milk, take 1ml sample into 2ml centrifuge tube(note: do not take the up-layer cream);

3) Add 50ul 1M HCl, shake strongly for 1min(or oscillator for 30s);

4) Centrifuge at above 4000 r/min at room temperature (20 - 25 ℃) for 10 minutes;

5) Take up-layer clear liquid 50ul into another clean centrifuge tube(note: do not take the up-layer cream), add 450ul diluted redissolving solution, shake strongly for 1min(or oscillator for 30s);

6) Take 50ul for analysis immediately.

Fold of dilution of the sample: 10

Milk (method 2)

1) Take 50ul liquid sample into 1950ul diluted redissolving solution; oscillator fully for 1min evenly;

2) Take 50ul for analysis immediately.

Fold of dilution of the sample: 40

5.4 Feed (Cattle feed, pig feed)

1. Take 1± 0.01 g feed sample into 50 mL centrifuge tube, add 5 mL deionized water, shake with oscillator for 2min until feed separate completely, centrifuge at above 4000 r/min for 10 minutes.
2. Take 40ul up-layer clear liquid into a new centrifuge tube, add 1560ul diluted redissolving solution, shake with oscillator for 30s;
3. Take 50 μL for analysis.

Fold of dilution of the sample: 200

6. ELISA procedures

Instructions

1. Bring all reagents and micro-well strips to the room temperature (20-25 ℃).
2. Return all reagents to 2-8 ℃ immediately after use.
3. The reproducibility of the ELISA analysis, to a large degree, depends on the consistency of plate washing. The correct operation of plate washing is the key point in the procedures of ELISA.
4. For the incubation at constant temperatures, all the samples and reagents must avoid light exposure, and each microplate should be sealed by the cover membrane.

Operation procedures

1. Take out all the necessary reagents from the kit and place at the room temperature (20 to 25 ℃) for at least 30 minutes. Note that each liquid reagent must be shaken to mix evenly before use.
2. Dilute the 5 concentrated standard solution separately: take 5 pieces of 2ml centrifuge tube, mark 0、0.4、1.2、3.6、10.8ppb accordingly, add 900μL the diluted redissolving solution into each tube, then add the five 10X concentrated standard solution into above 5 tubes accordingly, 100ul/tube. The 5 diluted standard solutions will be: 0—0、0.4—4、1.2—12、3.6—36、10.8—108.
3. Take the required micro-well strips and plate frames. Re-sealed the unused microplate, stored at 2-8 ℃, not frozen.
4. Numbering: number the micro-wells according to samples and standard solution; each sample and standard solution should be performed in duplicate; record their positions.
5. Add 50μL of the sample or standard solution to separate duplicate wells, then add enzyme conjugate, 50 μL each well, then add 50 μL of the antibody solution into each well. Mix gently by shaking the plate manually, seal the microplate with the cover membrane, and **incubate at 25 ℃ at dark for 30 minutes**.
6. Pour liquid out of microwell, add 250 μL/well of washing buffer for 15-30 seconds, repeat three to four times, then flap to dry (if there are the bubbles after flapping, cut them with the clean tips).
7. Coloration: add 100 μL mixture of the substrate A and substrate B into each well (Note: mix Substrate A and Substrate B at 1:1, the mixture should be used in 10min, never use metal container or metal to stir the solution, otherwise the substrate may be invalid.). Mix gently by shaking the plate manually, and **incubate at 25 ℃ for 15 minutes at dark for coloration.**
8. Determination: add 50 μL of the stop solution into each well (The substrate color from blue to yellow, it means the stop succeeds). Mix gently by shaking the plate manually. Set the wavelength of the microplate reader at 450 nm to determine the OD value (Recommend to read

the OD value at the dual-wavelength 450/630 nm within 5 minutes).

7. Result judgment

There are two methods to judge the results; the first one is the rough judgment, while the second is the quantitative determination. Note that the OD value of the sample has a negative correlation with the content of Oxytetracyline in the sample.

7.1 Qualitative determination

The concentration range (ng/mL) can be obtained from the comparison the average OD value of the sample with that of the standard solution. Assuming that the OD value of the sample Ⅰ is 0.3, and that of the sample Ⅱ is 1.0, while those of the standard solutions are as the followings: 2.243 for 0ppb, 1.816 for 0.4ppb, 1.415 for 1.2ppb, 0.74 for 3.6ppb, 0.313 for 10.8ppb, accordingly the concentration range of the sample Ⅰ is 3.6 to 10.88ppb, and that of the sample Ⅱ is 0.4 to 1.2 ppb.

7.2 Quantitative determination

The mean values of the absorbance values is equivalent to the percentage of the average OD value (B) of the sample and the standard solution divided by the OD value (B_0) of the first standard solution (0 standard) and subsequently multiplied by 100%, that is,

$$\text{Percentage of absorbance value} = \frac{B}{B_0} \times 100\%$$

B—the average (double wells) OD value of the sample or the standard solution

B_0—the average OD value of the 0 ng/mL standard solution

Draw the standard curve with the absorption percentages of the standard solutions and the semilogarithm values of the Oxytetracyline standard solutions (ng/mL) as Y- and X-axis, respectively. Read the corresponding concentration of the sample from the standard curve by incorporating its absorption percentage into the standard curve. The resulting value is subsequently multiplied by the corresponding dilution fold, thus finally obtaining Oxytetracyline concentration in the sample.

8. Precautions

1. The room temperature below 25 ℃ or the temperature of the reagents and the samples being not returned to the room temperature (20-25 ℃) will lead to a lower standard OD value.
2. Dryness of the microplate in the washing process will be accompanied by the situations including the non-linear standard curves and the undesirable reproducibility.
3. Mix every reagent and reaction mixture evenly and wash the microplate thoroughly, otherwise there will be the undesirable reproducibility.
4. The stop solution is the 2 M sulfuric acid solution, avoid contacting with the skin.
5. Put the unused microplate into an auto-sealing bag to re-seal it. The standard substance and the colourless color former is light sensitive, and thus they cannot be directly exposed to the light.
6. Do not use the kit exceeding its expiry date. The use of diluted or adulterated reagents from the kits will lead to the changes in the sensitivity and the detecting OD values. Do not exchange the

reagents from the kits of different lot numbers to use.

7. Discard the colouration solution with any color that indicates the degeneration of this solution. The detecting value of the 0 standard solution of less than 0.5 indicates its degeneration.
8. The optimum reaction temperature is 25 ℃, and too high or too low temperatures will result in the changes in the detecting sensitivity and OD values.

9. Storage and expiry date

Storage: store at 2-8 ℃, not frozen.

Expiry date: 12 months; date of production is on the box.

Note: If the Vacuum package of microplate has leakage, it is still valid to use, do not affect the test result, be relax to use.

Shenzhen Lvshiyuan Biotechnology Co., Ltd
D Building, National Biological Industrial Park of Marinelife, No.2 Binhai Road, Dapeng, Shenzhen, 518120 China
Tel. 86-755-28438788
Fax 86-755-28938800
Email: info@lsybt.com
www.lsybt.com

Tylosin ELISA Test Kit

Catalog No. LSY-10020

1. Principle

This test kit is based on the competitive enzyme immunoassay for the detection of Tylosin in the sample. The coupling antigens are pre-coated on the micro-well stripes. The Tylosin in the sample and the coupling antigens pre-coated on the micro-well stripes compete for the anti-Tylosin antibodies. After the addition of the enzyme conjugate, the TMB substrate is added for coloration. The optical density (OD) value of the sample has a negative correlation with the Tylosin in it. This value is compared to the standard curve and the Tylosin concentration is subsequently obtained.

2. Technical specifications

Sensitivity: 1ppb

Detection limit

Pork, Chicken (method 1)........................ about 20ppb

Pork, Chicken (method 2)........................ about 1ppb

Honey .... about 1ppb

Raw milk, reduced milk .... about 15ppb

Note: ppb= ng/ml or ng/g

Cross-reaction rate

Tylosin........................ 100%

Recovery rate

90±30%

3. Components

1) Micro-well strips: 12 strips with 8 removable wells each
2) 5× standard solution (1 mL each): 0ppb, 1ppb, 3ppb, 9ppb, 27ppb
3) Enzyme conjugate (12 mL)........................ 1 bottle
4) Antibody working solution (7mL)........................ 1 bottle
5) Substrate A (7 mL)........................ 1 bottle
6) Substrate B (7 mL)........................ 1 bottle
7) Stop solution (7 mL)........................ 1 bottle
8) 20× concentrated washing buffer (30 mL)........................ 1 bottle
9) 5× concentrated redissolving solution (10 mL)........................ 1 bottle

4. Materials required but not provided

1) **Equipments:** microplate reader (450nm, 630nm), printer, homogenizer, nitrogen-drying device, vortex, shaker, centrifuge (4000g and above), measuring pipets, balance (a reciprocal sensibility of 0.01 g), incubator (25℃), water bath, timer;
2) **Micropipettors:** single-channel 20-200 μL, 100-1000 μL, and eight-channel 30～300 μl;
3) **Reagents (AR):** 12MHCl, NaOH, ethyl acetate, n-Hexane, deionized water, $Na_2HPO_4.12H_2O$, $NaH_2PO4.2H_2O$.

5. Sample pre-treatment

Instructions

The following points must be dealt with before the pre-treatment of any kind of sample:

1）Only the disposable tips can be used for the experiments and the tips must be changed when used for absorbing different reagents;

2）Before the experiment, each experimental equipment must be clean and should be re-cleaned if necessary, in order to avoid the contamination that interferes with the experimental results.

Solution preparation before sample pre-treatment:

1) **Redissolving solution**: 1 part 5× concentrated redissolving solution + 4 parts deionized water.
2) **0.2M Phosphate buffer:** weigh 51.6g $Na_2HPO_4.12H_2O$ and 8.7g $NaH_2PO4.2H_2O$, add 1L deionized water to dissolve evenly..
3) **Washing buffer**: 1 part 20× concentrated washing buffer + 19 parts deionized water.
4) **0.1 M NaOH**: dissolve 0.8g NaOH in 200ml deionized water.
5) **1M HCl**: Weigh 1ml 12MHCl, add 11ml deionized water to dissolve.

5.1 Samples preparation

a) Chicken, pork (method 1)

1) Weigh 1± 0.05 g homogenized tissue sample into 50ml plastic centrifuge tube;
2) Add 2ml deionized water, shake strongly for 2min (or use vortex for 1min);
3) Centrifuge at above 4000 g at room temperature for 10min;
4) Take 100ul supernatant, add 400ul Redissolving solution, mix evenly for 10s;
5) Take 50ul to test.

Fold of dilution of the sample: 15

Chicken, pork (method 2)

1) Weigh 1± 0.05 g homogenized tissue sample into 50ml plastic centrifuge tube, add 1ml 0.2M Phosphate buffer to dissolve completely, then add 8ml ethyl acetate, shake or vortex for 3min, mix it evenly;

2) Centrifuge at above 3000 g at room temperature for 5min;

3) Take 4ml supernatant into a 10ml clean glass centrifuge tube, blow to dry in 50-60℃ water bath by nitrogen-drying device;

4) Add 1ml n-Hexane, vortex for 30s, then add 0.5ml Redissolving solution, vortex for 1min, mix evenly, centrifuge at above 3000 g at room temperature for 5min;

5) Discard up-layer organic phase, take 50ul down-layer liquid to test.

Fold of dilution of the sample: 1

b) Honey

1) Weigh 2± 0.05 g honey sample into 50ml plastic centrifuge tube, add 2ml 0.1M NaOH to dissolve completely, then add 6ml ethyl acetate, shake for 5min, mix evenly;

2) Centrifuge at above 3000 g at room temperature for 5min;

3) Take 3ml up-layer organic phase into 10ml clean dry glass tube, blow to dry in 50-60℃ water bath by nitrogen-drying device;

4) Add 0.5ml Redissolving solution, vortex for 1min, be static at room temperature for 5min, vortex for 1min again, mix evenly;

5) Take 50ul down-layer liquid to test.

Fold of dilution of the sample: 0.5

c) Raw milk, reduced milk

Reduced milk: weigh 1g±0.05g milk powder, add 7ml deionized water, shake and dissolve, become reduced milk.

1) Take the collected raw milk/reduced milk, thaw and return to room temperature for above 30min;
2) Put tips into down-layer of raw milk/reduced milk, take out 1ml sample into 2ml centrifuge tube(Note: do not take up-layer cream);
3) Add 50ul 1M HCl, shake strongly for 1min (or vortex for 30s);
4) Centrifuge at above 4000 g at room temperature for 10min;
5) Take 100ul up-layer clear liquid into another centrifuge tube (do not take up-layer cream), then add 900ul Redissolving solution,shake strongly for 1min (or vortex for 30s);
6) Take 50ul liquid to test.

Fold of dilution of the sample: 10

6. ELISA procedures

6.1 Instructions

1) Bring all reagents and micro-well strips to the room temperature (20-25 ℃) before use;
2) Return all reagents to 2-8 ℃ immediately after use;
3) The reproducibility of the ELISA analysis, to a large degree, depends on the consistency of plate washing. The correct operation of plate washing is the key point in ELISA the procedures;
4) For the incubation at constant temperatures, all the samples and reagents must avoid light exposure, and each microplate should be sealed by the cover membrane.

6.2 Operation procedures

1. Bring test kit to the room temperature (20-25 ℃) for at least 30 min, note that each reagent must be shaken to mix evenly before use, put the required micro-well strips into plate frames. Re-sealed the unused microplate, store at 2-8 ℃, not frozen.
2. Numbering: number the micro-wells according to samples and standard solution; each sample and standard solution should be performed in duplicate, record their positions.
3. Add 50 μL of the sample or standard solution into separate duplicate wells; then add 50 μL of the antibody working solution into each well, mix gently by shaking the plate manually. Seal the microplate with the cover membrane, and **incubate at 25 ℃ for 30min in dark.**
4. Pour liquid out of microwell, flap to dry on absorbent paper, add 250 μL/well of washing buffer to wash microplate for 15-30 s, then take out and flap to dry with absorbent paper, repeat 3-4 times. (If there are the bubbles after flapping, cut them with the clean tips).
5. Add 100ul enzyme conjugate, mix gently by shaking the plate manually. Seal the microplate with the cover membrane, and **incubate at 25 ℃ for 30min in dark.** Washing as step 4.
6. Coloration: add 100ul mixture of substrate A solution and substrate B solution into each well (Note: mix substrate A solution and substrate B solution at 1:1, use the mixture in 10min, do not use metal to contain or stir, to avoid substrate invalid). Mix gently by shaking the plate manually, seal the microplate with the cover membrane then **incubate at 25 ℃ for 15 min at dark for coloration.**

7. Determination: add 50 μL of the stop solution into each well. Mix gently by shaking the plate manually. Stop successfully when substrate color from blue to yellow. Recommend to read the OD value at the dual-wavelength 450/630 nm within 5 minutes.

7. Result judgment

There are two methods to judge the results: the first one is the rough judgment, while the second is the quantitative determination. Note that the OD value of the sample has a negative correlation with the Tylosin concentration.

7.1 Qualitative determination

The concentration range (ng/mL) of Tylosin can be obtained from comparing the average OD value of the sample with that of the standard solution. Assuming that the OD value of the sample Ⅰ is 0.3, and that of the sample Ⅱ is 1.0, the OD value of standard solutions is: 2.243 for 0ppb, 1.816 for 1ppb, 1.415 for 3ppb, 0.74 for 9ppb, 0.313 for 27ppb, accordingly the concentration range of the sample Ⅰ is 9 to 27ppb, and that of the sample Ⅱ is 1 to 3ppb.

7.2 Quantitative determination

The mean values of the absorbance values is obtained for the average OD value (B) of the sample and the standard solution divided by the OD value (B_0) of the first standard solution (0 standard) and subsequently multiplied by 100%, that is,

$$\text{Percentage of absorbance value} = \frac{B}{B_0} \times 100\%$$

B—the average OD value of the sample or the standard solution

B_0—the average OD value of the 0 ng/mL standard solution

Draw the standard curve with the absorption percentages of the standard solution and the semilogarithm values of the Tylosin standard solution (ng/mL) as Y- and X-axis, respectively. Read the corresponding concentration of the sample from the standard curve by incorporating its absorption percentage into the standard curve. The resulting value is subsequently multiplied by the corresponding dilution fold, finally obtaining the Tylosin concentration in the sample.

8. Precautions

1. The room temperature below 25 ℃ or the temperature of the reagents and the samples being not returned to the room temperature (20-25 ℃) will lead to a lower standard OD value.
2. Dryness of the microplate in the washing process will be accompanied by the situations including the non-linear standard curves and the undesirable reproducibility; So continue to next step immediately after washing.
3. Mix evenly, otherwise there will be the undesirable reproducibility.
4. The stop solution is the 2 M sulfuric acid solution, avoid contacting with the skin.
5. Do not use the kit exceeding its expiry date. The use of diluted or adulterated reagents from the kits will lead to the changes in the sensitivity and the detecting OD values. Do not exchange the reagents from the kits of different lots to use.
6. Put the unused microplate into an auto-sealing bag to re-seal it. The standard solution and the colourless color former is light sensitive, and thus they cannot be directly exposed to the light.

7. Discard the colouration solution with any color that indicates the degeneration of this solution. The detecting value of the standard solution 1(0 ppb) of less than 0.5 indicates its degeneration.

9. Storage and expiry date

Storage: store at 2-8 ℃, not frozen.

Expiry date: 12 months; date of production is on box.

Shenzhen Lvshiyuan Biotechnology Co., Ltd
D Building, National Biological Industrial Park of Marinelife, No.2 Binhai Road, Dapeng, Shenzhen, 518120 China
Tel. 86-755-28438788
Fax 86-755-28938800
Email: info@lsybt.com
www.lsybt.com

Green Spring

Shenzhen Lvshiyuan Biotechnology Co.,Ltd

Version：2016-01

Sulfamethazine (SM2) ELISA Kit

Catalog No. LSY-10010

1. Principle

This test kit is based on the indirect competitive enzyme immunoassay for the detection of Sulfamethazine (SM2) residue. The coupling antigens are pre-coated on the micro-well stripes. The Sulfamethazine in the sample and the coupling antigens pre-coated on the micro-well stripes compete for the anti-Sulfamethazine antibodies. After the addition of the enzyme conjugate, the TMB substrate is added for coloration. The optical density (OD) value of the sample has a negative correlation with the Sulfamethazine in the sample. This value is compared to the standard curve and the Sulfamethazine concentration is subsequently obtained.

2. Technical specifications

Sensitivity: 1 ppb

Incubator temperature: 25℃

Incubator time: 30min～15min

Detection limit

Tissue (high-detection-limit method)..

Tissue (lower-detection-limit method)……………………………………….1 ppb

Honey, egg..5 ppb

Serum, urine...1 ppb

Milk...4 ppb

Cross-reaction rate...20 ppb

Sulfamethazine(SM2)…………………………………………………………

Recovery rate...100%

Tissue, urine, milk..

Honey, serum, egg... 85±25%

....80±23%

3. Components

1	Micro-well strips	12 strips with 8 removable wells each	
2	6× standard solution (1mL each)	0ppb 1ppb<br>3ppb 9ppb<br>27ppb 81ppb	
3	Enzyme conjugate	7ml	red cap
4	Antibody working solution	7ml	blue cap
5	Substrate A	7ml	white cap
6	Substrate B	7ml	black cap
7	Stop solution	7ml	yellow cap
8	20× concentrated washing buffer	40ml	white cap
9	20×concentrated redissolving solution	50ml	transparent cap

4. Materials required but not provided

1) **Equipments:** microplate reader, printer, homogenizer, nitrogen-drying device, votex, centrifuge, measuring pipets, balance (a sensibility reciprocal of 0.01 g), Incubator
2) **Micropipettors:** single-channel 20-200 μL, 100-1000 μL, and multi-channel 30-300 μL;
3) **Reagents:** Acetonitrile (CH_3CN), ethyl acetate, N-hexane, $K_2HPO_4 \cdot 12H_2O$, Citric acid monohydrate ($C_6H_8O_7 \cdot H_2O$), HCl, NaOH, CH_2Cl_2,

5. Sample pre-treatment

Instructions (The following points must be dealt with before the pre-treatment)

1) Only the disposable tips can be used for the experiments and the tips must be changed when used for absorbing different reagents;
2) Before the experiment, each experimental utensil must be clean and should be re-cleaned if necessary, in order to avoid the contamination that interferes with the experimental results.

Solution preparation before sample pre-treatment:

1) 0.2M NaOH solution: Weigh 0.8g NaOH, dissolve with 100ml deionized water;
2) 0.5M HCl: Take 4.3ml HCl, dissolve with deionized water to 100ml, mix evenly;
3) Na_2HPO_4- $C_6H_8O_7 \cdot H_2O$ buffer: weigh 19.85g$Na_2HPO_4 \cdot 12H_2O$ and 9.3g $C_6H_8O_7 \cdot H_2O$, dissolve with deionized water to 1L, mix evenly;
4) CH_3CN-CH_2Cl_2 solution: V_{CH3CN}: V_{CH2Cl2} =1:4 ;
5) The 20× concentrated redissolving solution is diluted with deionized water at 1:19(1 part concentrated redissolbing solution + 19 parts deionized water).

5.1 Tissue

A. High-detection-limit method

Method one

1) Weigh 2.0 ± 0.05 g of the homogenized tissue sample into 50 ml centrifuge tube, add 6 ml ethyl acetate, shake for 2 min, centrifuge at above 4000 r/min at 15 ℃ for 10 min;
2) Take 3 ml the clear organic phase into a dry container, blow to dry with nitrogen or air completely by rotary evaporation at 50-60 ℃
6) Dissolve the dry residues in 1 mL of the diluted redissolving solution, add 1 mL N-hexane, mix for 30 seconds; centrifuge at above 4000 r/min at 15℃ for 5 min. Remove the upper layer N-hexane phase,
7) Take down-layer 50μl solution for further analysis.

Fold of dilution of the sample: 1

Method two (for tissue and egg)

1) Weigh 2 ± 0.05 g of the homogenized sample, put into 50ml centrifugal tube;
2) Add 8ml CH_3CN-CH_2Cl_2 solution, shake for 5min, centrifuge at above 4000 r/min at 15 ℃ for 10 min;
3) Transfer 4 ml organic phase into a dry container, blow to dry with nitrogen or air completely by rotary evaporation at 56 ℃
4) Add 1 mL of the diluted redissolving solution to redissolve the dry residue, add 1 mL N-hexane, shake for 30s. Centrifuge at above 4000 r/min at 15℃ for 5 min;
5) Remove the upper layer N-hexane phase. Take 50 μL down layer solution for further analysis.

Fold of dilution of the sample: 1

B. Tissue lower-detection-limit method

1) Weigh 2.0 ± 0.05 g of the homogenized sample into a 50 ml centrifugal tube, add 8 mL diluted redissolving solution, shake for 2 min, centrifuge at above 4000 r/min at 15 ℃ for 10 min;
2) Take 50 μL solution for further analysis.
   Fold of dilution of the sample: 5

5.2 Serum

1) Place the serum sample in the room temperature for 30 min, centrifuge at above 4000r/min at 10 ℃ for 10 min, separation of the serum or filter serum
2) Take 1 mL serum and add 3mL the diluted redissolving solution, mix for 30s.
3) Take 50 μL solution for further analysis
   Fold of dilution of the sample: 4

5.3 Honey

1. Weight 1 ± 0.05 g honey sample into 50 mL centrifugal tube, add 1ml 0.5M HCl solution, put it into 37℃ environment for 30min;
2. Add 2.5ml 0.2M NaOH solution and 3ml Na_2HPO_4- $C_6H_8O_7 \cdot H_2O$ buffer separately, then add 4ml ethyl acetate, shake for 2min, centrifuge at above 4000 r/min at 15 ℃ for 10 min;
3. Take 2mL up-layer organic phase, blow to dry with nitrogen at 50-60 ℃, add 0.5 mL of the diluted redissolving solution to redissolve, mix for 30s.
4. Take 50 μL for further analysis
   Fold of dilution of the sample: 1

5.4 Urine

1. Add 3 mL the diluted redissolving solution and 1 mL of the centrifuged clear urine sample, mix properly for 30s.
2. Take 50 μL for further analysis
   Fold of dilution of the sample: 4

5.5 Milk

1. Take 1 mL milk, add the diluted redissolving solution, dilute at 1:19(V/V) (20 μL milk + 380 μL the diluted redissolving solution), mix for 30s.
2. Take 50 μL for further analysis
   Fold of dilution of the sample: 20

6. ELISA procedures

6.1 Instructions

1. Bring all reagents and micro-well strips to the room temperature (20-25 ℃) before use;
2. Return all reagents to 2-8 ℃ immediately after use;
3. The reproducibility of the ELISA analysis, to a large degree, depends on the consistency of plate washing. The correct operation of plate washing is the key point in the procedures of ELISA;
4. For the incubation at constant temperatures, all the samples and reagents must avoid light exposure, and each microplate should be sealed by the cover membrane.

6.2 Operation procedures

1. Take out all the necessary reagents and place at the room temperature (20-25 ℃) for at least 30min. Note that each reagent must be shaken to mix evenly before use;
2. Take the required micro-well strips and plate frames. Re-sealed the unused microplate, stored

at 2- 8 ℃;

3. Washing buffer preparation: dilute 40 mL of the 20× concentrated washing buffer with the deionized water at 1:19 (1 part 20× concentrated washing buffer + 19 parts deionized water). Or prepare washing buffer as quantity needed.
4. Numbering: number the micro-wells according to samples and standard solution; each sample and standard solution should be performed in duplicate; record their positions;
5. Add 50 μL of the sample or standard solution to separate duplicate wells, add 50 μL of enzyme conjugate, then add 50 μL of the antibody working solution into each well. Mix by shaking gently, seal the microplate with the cover membrane, **and incubate at 25℃ for 30 min**;
6. Wash the microplate with the washing buffer at 250 μL/well for four to five times.;soak the well with the washing buffer for 15-30 sec, flap to dry with absorbent paper (if there are the bubbles after flapping, cut them with the clean tips);
7. Coloration: add 50 μL of the substrate A solution and 50 μL of the B solution into each well. Mix by shaking gently, **and incubate at 25 ℃ for 15 min in the dark for coloration**;
8. Determination: add 50 μL of stop solution into each well. Mix by shaking gently. Set the wavelength of the microplate reader at 450 nm to determine the OD value. (Recommend to read the OD value at the dual-wavelength 450/630 nm within 5 min) **.**

7. Result judgment

There are two methods to judge the results; the first one is the rough judgment, while the second is the quantitative determination. Note that the OD value of the sample has a negative correlation with the content of Sulfamethazine in the sample.

7.1 Qualitative determination

The concentration range (ng/mL) obtained from the comparison the average OD value of the sample with that of the standard solution. Assuming that the OD value of the sample Ⅰ is 0.3, and that of the sample Ⅱ is 1.0, the OD value of standard solutions is: 2.243 for 0ppb, 1.816 for 1ppb, 1.415 for 3ppb, 0.74 for 9ppb, 0.313 for 27ppb, 0.155 for 81ppb, accordingly the concentration range of the sample Ⅰ is 27ppb to 81ppb, and that of the sample Ⅱ is 3ppb to 9ppb. (multiplied by the corresponding dilution fold)

7.2 Quantitative determination

The mean values of the absorbance values is equivalent to the percentage of the average OD value (B) of the sample and the standard solution divided by the OD value (B_0) of the first standard solution (0 standard) and subsequently multiplied by 100%, that is,

$$\text{Percentage of absorbance value} = \frac{B}{B_0} \times 100\%$$

B—the average (double wells) OD value of the sample or the standard solution

B_0—the average OD value of the 0ng/mL standard solution

Draw the standard curve with the absorption percentages of the standard solutions and the semilogarithm values of the Sulfamethazine standard solutions (ng/mL) as Y- and X-axis, respectively. Read the corresponding concentration of the sample from the standard curve by incorporating its absorption percentage into the standard curve. The resulting value is subsequently multiplied by the corresponding dilution fold, finally obtaining the Sulfamethazine concentration in

the sample.

Using the professional analyzing software of this kit will be more convenient for the accurate and rapid analysis of a large amount of samples. (Please contact us for this software)

8. Precautions

1. The room temperature below 25 ℃ or the temperature of the reagents and the samples being not returned to the room temperature (20-25 ℃) will lead to a lower standard OD value.
2. Dryness of the microplate in the washing process will be accompanied by the situations including the non-linear standard curves and the undesirable reproducibility; So continue to next step immediately after washing.
3. Mix evenly, otherwise there will be the undesirable reproducibility.
4. The stop solution is the 2 M sulfuric acid solution, avoid contacting with the skin.
5. Do not use the kit exceeding its expiry date. The use of diluted or adulterated reagents from the kits will lead to the changes in the sensitivity and the detecting OD values. Do not exchange the reagents from the kits of different lot numbers to use.
6. Put the unused microplate into an auto-sealing bag to re-seal it. The standard substance and the colourless color former is light sensitive, and thus they cannot be directly exposed to the light.
7. Discard the colouration solution with any color that indicates the degeneration of this solution. The detecting value of the 0 standard solution of less than 0.5 (A450 nm< 0.5) indicates its degeneration.
8. The optimum reaction temperature is 25 ℃, and too high or too low temperatures will result in the changes in the detecting sensitivity and OD values.

9. Storage and expiry date

Storage: store at 2-8 ℃, not frozen.

Expiry date: 1 year; date of production is on box.

Note: If the Vacuum package of microplate has leakage, it is still valid to use, do not affect the test result, be relax to use.

Shenzhen Lvshiyuan Biotechnology Co., Ltd
D Building, National Biological Industrial Park of Marinelife, No.2 Binhai Road, Dapeng, Shenzhen, 518120 China
Tel. 86-755-28438788
Fax 86-755-28938800
Email: info@lsybt.com
www.lsybt.com

Gentamicin ELISA Test Kit

Catalog No. LSY-10023

1. Principle

This test kit is based on the competitive enzyme immunoassay for the detection of Gentamicin in the sample. The coupling antigen is pre-coated on the micro-well stripes. The Gentamicin in the sample and pre-coated coupling antigen on the micro-well stripes compete for the anti-Gentamicin antibody. After the addition of the enzyme conjugate, the TMB substrate is added for coloration. The optical density (OD) value of the sample has a negative correlation with the Gentamicin in it. The value is compared to the standard curve and the Gentamicin concentration is subsequently obtained.

2. Technical specifications

Sensitivity: 0.05 ppb

Incubator temperature: 25℃

Incubator time: 30min～15min

Detection limit

Tissue.. 2.5 ppb
Liver, milk.. 3 ppb

Recovery rate

Tissue.. 90±20%
Liver... 80±20%
Milk.. 90±18%

Cross-reaction rate

Gentamicin.. 100%
Streptomycin.. <1%
Dihydrostreptomycin.. <1%
Neomycin... <1%

3. Components

1	Micro-well strips	12 strips with 8 removable wells each	
2	6× standard solution (1 mL each)	0ppb 0.05ppb<br>0.15ppb 0.45ppb<br>1.35ppb 4.05ppb	
3	Enzyme conjugate	7ml	red cap
4	Antibody working solution	7ml	blue cap
5	Substrate A	7ml	white cap
6	Substrate B	7ml	black cap
7	Stop solution	7ml	yellow cap
8	20× concentrated washing buffer	15ml	white cap
9	Sample redissolving solution	50ml	transparent cap
10	10× concentrated extracting solution	50ml	blue cap

4. Materials required but not provided

1) **Equipments:** microplate reader, votex, centrifuge, homogenizer, measuring pipets, balance (a sensibility reciprocal of 0.01 g).
2) **Micropipettors:** single-channel 20-200 μL and 100-1000 μL, and multi-channel 30～300 μL;

5. Sample pre-treatment

Instructions

The following points must be dealt with before the pre-treatment of any kind of sample:

1) Only the disposable tips can be used for the experiments and the tips must be changed when used for absorbing different reagents;
2) Before the experiment, each experimental utensil must be checked to be clean and should be re-cleaned if necessary, in order to avoid the contamination that interferes with the experimental results.

Solution preparation before sample pre-treatment:

Dilute the 10× concentrated extracting solution with the deionized water at 1:9 (1 part concentrated extracting solution + 9 parts deionized water)

5.1 Tissue (Chicken, pork, fish, shrimp) and Liver(Chicken liver, porcine liver)

1. Take 1±0.05g of the sample, which already crumble and remove fat, add 5mL diluted extracting solution, shake for 3min; centrifuge at 4000r/min at room temperature (20-25 ℃) for 10min.
2. Take 50μL supernatant (upper layer), add 450μL of the Sample redissolving solution, mix for 30s.
3. Take 50 μL for analysis.

Fold of dilution of the sample: 50

5.2 Milk

1. Take 0.5ml homogeneous milk sample, add 2ml diluted extracting solution, shake for 2min; centrifuge at 4000r/min at room temperature (20-25 ℃) for 5min.
2. Take 50μL supernatant (upper layer), add 450μL of the Sample redissolving solution, mix for 30s.
3. Take 50 μL for analysis.

Fold of dilution of the sample: 50

6. ELISA procedures

6.1 Instructions

1 Bring all reagents and micro-well strips to the room temperature (20-25 ℃) before use.
2 Return all reagents to 2-8 ℃ immediately after use.
3 The reproducibility of the ELISA analysis, to a large degree, depends on the consistency of plate washing. The correct operation of plate washing is the key point in the procedures of ELISA.
4 For the incubation at constant temperatures, all the samples and reagents must avoid light exposure, and each microplate should be sealed by the cover membrane.

6.2 Operation procedures

1. Take out the kit from the refrigerated environment. Take out all the necessary reagents from the

kit and place at the room temperature (20-25 ℃) for at least 30 min. Note that each liquid reagent must be shaken to mix evenly before use.

2. Take the required micro-well strips and plate frames. Re-sealed the unused microplate, stored at 2-8 ℃, not frozen.
3. Solution preparation: dilute 15mL of the 20X concentrated washing buffer with deionized water at 1:19 (1 part 20X concentrated washing buffer + 19 parts deionized water), or dilute quantity as required.
4. Numbering: number the micro-wells according to samples and standard solution; each sample and standard solution should be performed in duplicate; record their positions.
5. Add 50μL of the sample or standard solution to separate duplicate wells, and add 50μL enzyme conjugate and then 50μL of the antibody working solution into each well. Mix gently by shaking the plate manually, seal the microplate with the cover membrane, **and incubate at 25 ℃ for 30 min.**
6. Pour liqiud out of the microwells, add 250 μL/well of diluted washing buffer for 15-30 sec, repeat 4-5 times, then flap to dry with absorbent paper (if there are the bubbles after flapping, cut them with the clean tips).
7. Coloration: add 50μL of the substrate A and then 50μL of the substrate B into each well. Mix gently by shaking the plate manually, and **incubate at 25 ℃ for 15 min** at dark for coloration;
8. Determination: add 50μL of the stop solution into each well. Mix gently by shaking the plate manually. Set the wavelength of the microplate reader at 450nm to determine the OD value (Recommend to read the OD value at the dual-wavelength 450/630nm within 5 min).

7. Result judgment

There are two methods to judge the results; the first one is the rough judgment, while the second is the quantitative determination. Note that the OD value of the sample has a negative correlation with the content of Gentamicin.

7.1 Qualitative determination

The concentration range (ng/mL) can be obtained from the comparison the average OD value of the sample with that of the standard solution. Assuming that the OD value of the sample Ⅰ is 0.3, and that of the sample Ⅱ is 1.0, while those of the standard solutions are as the followings: 2.243 for 0ppb, 1.816 for 0.05ppb, 1.415 for 0.15ppb, 0.74 for 0.45ppb, 0.313 for 1.35ppb and 0.155 for 4.05ppb, accordingly the concentration range of the sample Ⅰ is 1.35 to 4.05ppb, and that of the sample Ⅱ is 0.15 to 0.45ppb. (multiplied by the corresponding dilution fold).

7.2 Quantitative determination

The mean values of the absorbance values is equivalent to the percentage of the average OD value (B) of the sample and the standard solution divided by the OD value (B_0) of the first standard solution (0 standard) and subsequently multiplied by 100%, that is

$$\text{Percentage of absorbance value} = \frac{B}{B_0} \times 100\%$$

B—the average (double wells) OD value of the sample or the standard solution

B_0—the average OD value of the 0ng/mL standard solution.

Draw the standard curve with the absorption percentages of the standard solutions and the semilogarithm values of the Gentamicin standard solutions (ng/mL) as Y- and X-axis, respectively. Read the corresponding concentration of the sample from the standard curve by incorporating its absorption percentage into the standard curve. The resulting value is subsequently multiplied by the corresponding dilution fold, thus finally obtaining the Gentamicin concentration in the sample.

8. Precautions

1. The room temperature below 25 ℃ or the temperature of the reagents and the samples being not returned to the room temperature (20-25 ℃) will lead to a lower standard OD value.
2. Dryness of the microplate in the washing process will be accompanied by the situations including the non-linear standard curves and the undesirable reproducibility.
3. Mix every reagent and reaction mixture evenly and wash the microplate thoroughly, otherwise there will be the undesirable reproducibility.
4. The stop solution is the 2 M sulfuric acid solution, avoid contacting with the skin.
5. Put the unused microplate into an auto-sealing bag to re-seal it. The standard substance and the colourless color former is light sensitive, and thus they cannot be directly exposed to the light.
6. Do not use the kit exceeding its expiry date. The use of diluted or adulterated reagents from the kits will lead to the changes in the sensitivity and the detecting OD values. Do not exchange the reagents from the kits of different lot numbers to use.
7. Discard the colouration solution with any color that indicates the degeneration of this solution. The detecting value of the 0 standard solution of less than 0.5 indicates its degeneration.
8. The optimum reaction temperature is 25 ℃, and too high or too low temperatures will result in the changes in the detecting sensitivity and OD values.

9. Storage and expiry date

Storage: store at 2-8 ℃, not frozen.

Expiry date: 12 months; date of production is on the box.

Note: If the Vacuum package of microplate has leakage, it is still valid to use, do not affect the test result, be relax to use.

Shenzhen Lvshiyuan Biotechnology Co., Ltd

D Building, National Biological Industrial Park of Marinelife, No.2 Binhai Road, Dapeng, Shenzhen, 518120 China

Tel. 86-755-28438788

Fax 86-755-28938800

Email: info@lsybt.com

www.lsybt.com
